# The mitogenome of the crab *Grapsus adscensionis* (Osbeck, 1765)

**DOI:** 10.1080/23802359.2025.2571726

**Published:** 2025-10-16

**Authors:** Cátia Alves, Manuel Curto, Lara Baptista, Thapasya Vijayan, Livia Sinigaglia, Patrícia Madeira, António Santos, Harald Meimberg, Manuel Lopes-Lima, Sérgio P. Ávila

**Affiliations:** a CIBIO, Centro de Investigação em Biodiversidade e Recursos Genéticos, InBIO Laboratório Associado, Ponta Delgada, Portugal; b BIOPOLIS Program in Genomics, Biodiversity and Land Planning, CIBIO, Vairão, Portugal; c UNESCO Chair – Land Within Sea: Biodiversity & Sustainability in Atlantic Islands, Universidade dos Açores, Ponta Delgada, Portugal; d Faculdade de Ciências da Universidade do Porto, Porto, Portugal; e MPB – Marine Palaeontology and Biogeography Lab, Universidade dos Açores, Ponta Delgada, Portugal; f CIBIO, Centro de Investigação em Biodiversidade e Recursos Genéticos, InBIO Laboratório Associado, Universidade do Porto, Vairão, Portugal; g Department of Ecosystem Management, Climate and Biodiversity, Institute of Integrative Nature Conservation Research, BOKU University, Vienna, Austria; h Royal Netherlands Institute for Sea Research, Texel, Netherlands

**Keywords:** Brachyura, Grapsidae, mitochondrial genome

## Abstract

The mitochondrial genome of *Grapsus adscensionis* (Osbeck, 1765), a rocky shore crab distributed across the Macaronesian archipelagos and the eastern Atlantic, was sequenced and annotated for the first time. The circular mitogenome is 15,553 bp in length and comprises 13 protein-coding genes (PCGs), 22 tRNAs, two rRNAs, and a non-coding control region. It exhibits a strong AT bias, with negative AT- and GC-skews. Gene arrangement and composition are consistent with other brachyuran crabs. These findings provide novel molecular data for the species, representing the third mitogenome published for the genus *Grapsus*, and contribute to a better understanding of brachyuran evolutionary relationships.

## Introduction

*Grapsus adscensionis* (Osbeck, 1765) is a rocky shore crab widely distributed throughout the Macaronesian archipelagos. It is also reported from the islands of Ascension and Saint Helena, and it occurs as well along parts of the eastern Atlantic coastline (Araújo and Calado 2003; Freire et al. 2021). This species is ecologically significant, occupying intertidal habitats where it plays a role in nutrient cycling and shoreline ecosystem dynamics (Ramírez and Haroun 2014). Despite its ecological relevance, no complete mitochondrial genome has been published to date for the species. Therefore, the present study provides the first complete and annotated mitochondrial genome of *G. adscensionis.* This genomic resource will support future comparative genomic and phylogenetic studies and help clarify evolutionary patterns within Grapsidae.

## Materials and methods

**Figure 1.**
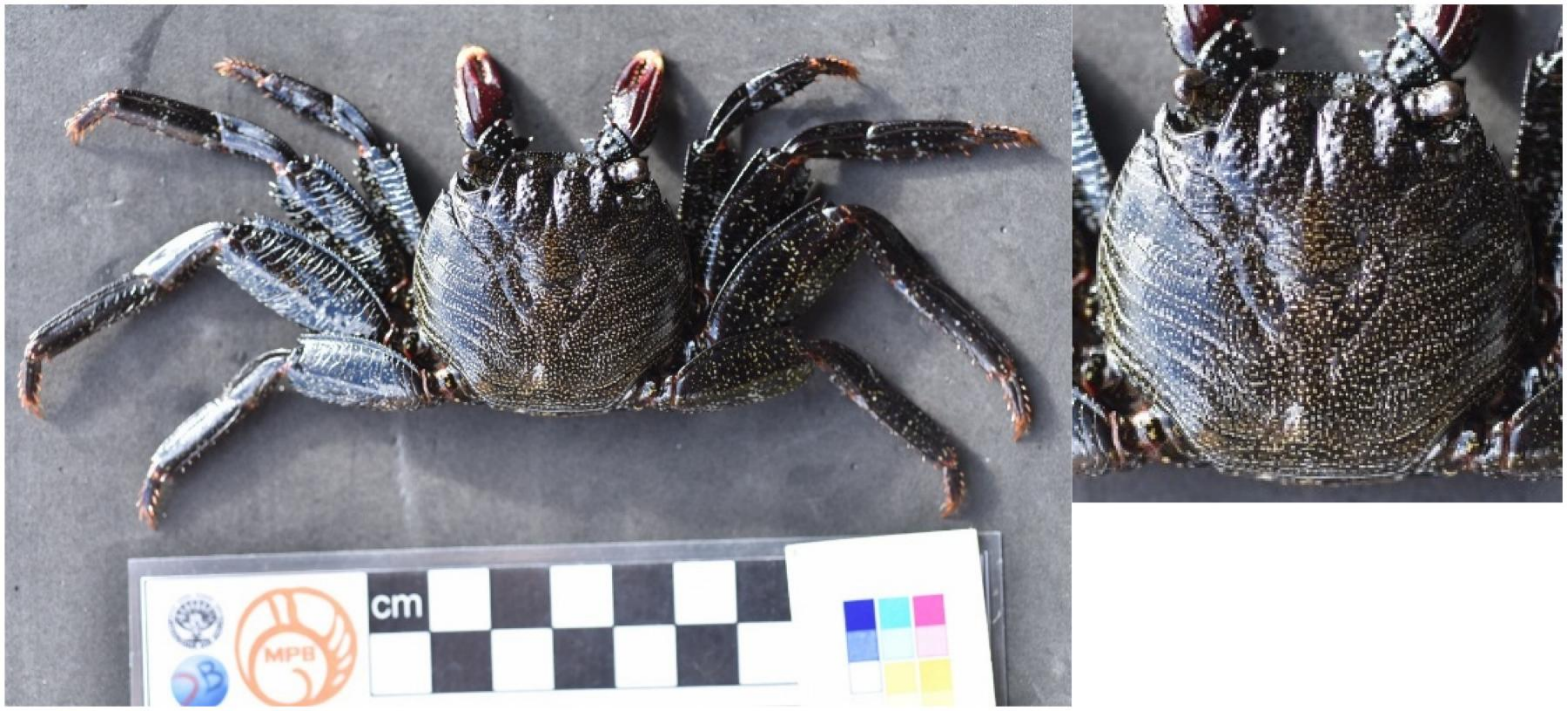
Photograph of *G. adscensionis* collected at Pico Island (photograph taken by Cátia Alves for this work). This species differs from the only congeneric species in the Atlantic Ocean, *Grapsus grapsus* (Linnaeus, 1758), by its rounder shape and dotted ornamentation of the cephalothorax (Freire et al. 2021).

A low-coverage Illumina MiSeq (San Diego, CA) run was performed for one individual of *G. adscensionis* (Figure 1; specimen voucher DBUA-CRU-312 deposited in the collection of the Department of Biology of the University of the Azores – https://www.uac.pt; contact person Sérgio Ávila – avila@uac.pt) hand-captured at the intertidal of Pico Island (38.392537, −28.254605) in August 2021. The leg sample was preserved in ethanol 96% until DNA extraction following a SDS-based buffer tissue protocol, detailed in the Supplementary Materials. Library preparation and shotgun sequencing were carried out using an Illumina MiSeq (San Diego, CA) paired-end (PE) 300 bp run as a service provided by the Genomics Service Unit at Ludwig-Maximilian University Munich, Munich, Germany. The mitogenome was deposited in GenBank (https://www.ncbi.nlm.nih.gov/genbank/) under the accession number PV599762. The assemblage was done using Geneious Prime 2023.1.1 (read coverage depth map available as Figure S1); gene annotation was performed with MITOS2 (Bernt et al. 2013) and tRNA annotation with ARWEN v1.2 (Laslett and Canbäck 2008). Genome illustration was obtained with Proksee (Grant et al. 2023). The 13 coding proteins of the mitogenome (PCGs) of 19 crabs from the Thoracotremata group and two outgroups from Heterotremata: *Maja crispata* Risso, 1827 and *Calappa bilineata* Ng, Lai & Aungtonya, 2002 were included to assess the phylogenetic relationship among Brachyura. When data were available in GenBank, a single representative species per family was selected, except for the *Grapsus* genus. Phylogenetic tree reconstruction was performed in IQ-TREE 2 (Minh et al. 2020) using the maximum-likelihood (ML) method, with 1000 bootstrap replicates to assess node support (Felsenstein 1985). This was conducted under the TPM2u + F + I + G4 model of molecular evolution.

## Results

**Figure 2.**
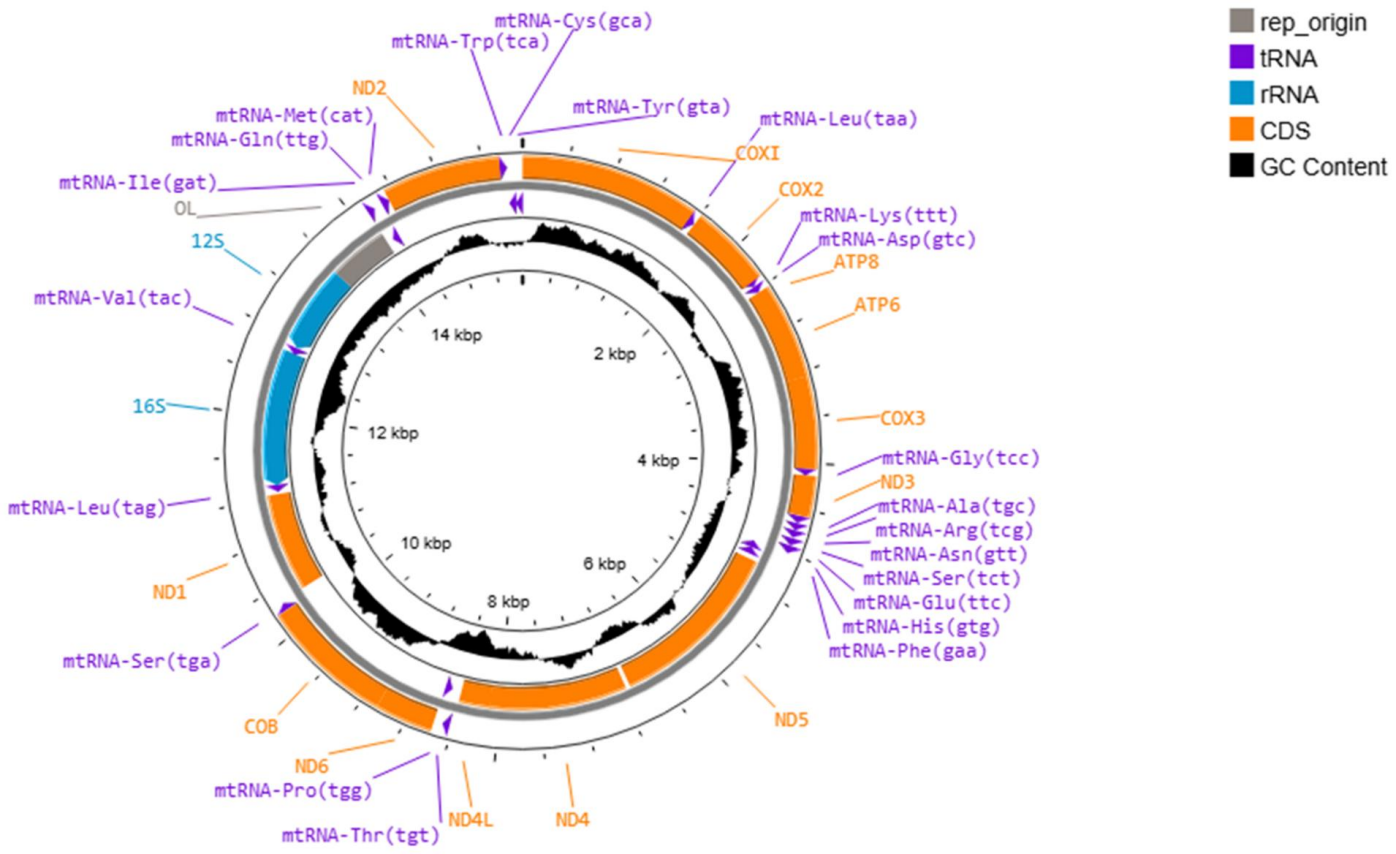
Map of the mitogenome of *G. adscensionis*. Clockwise (outer circle) and counterclockwise (inner circle) transcribed genes are a part of the heavy DNA strand (+) and light DNA strand (−), respectively. The GC contents are displayed in the center.

The mitogenome of *G. adscensionis* is a closed circular molecule with 15,553 bp in size (Figure 2), consistent with the mitochondrial genome organization of other decapod species. The nucleotide composition was skewed (A: 32.3%, T: 33.3%, G: 12%, and C: 22.4%), with a strong AT bias. The AT-skew (−0.015) and GC-skew (≈−0.303) for the whole mitogenome are both negative, indicating a higher occurrence of Ts and Cs rather than As and Gs, respectively. This genome includes 13 PCGs (cox1–3, nad1–6, nad4L, cob, atp6, and atp8), two rRNA genes (12S and 16S), 22 tRNA genes, and a major non-coding region known as the CR (Table S1). Of the 37 annotated genes, only 14 are encoded in the light strand. Three types of start codons were observed throughout PCGs (Table S1).

**Figure 3.**
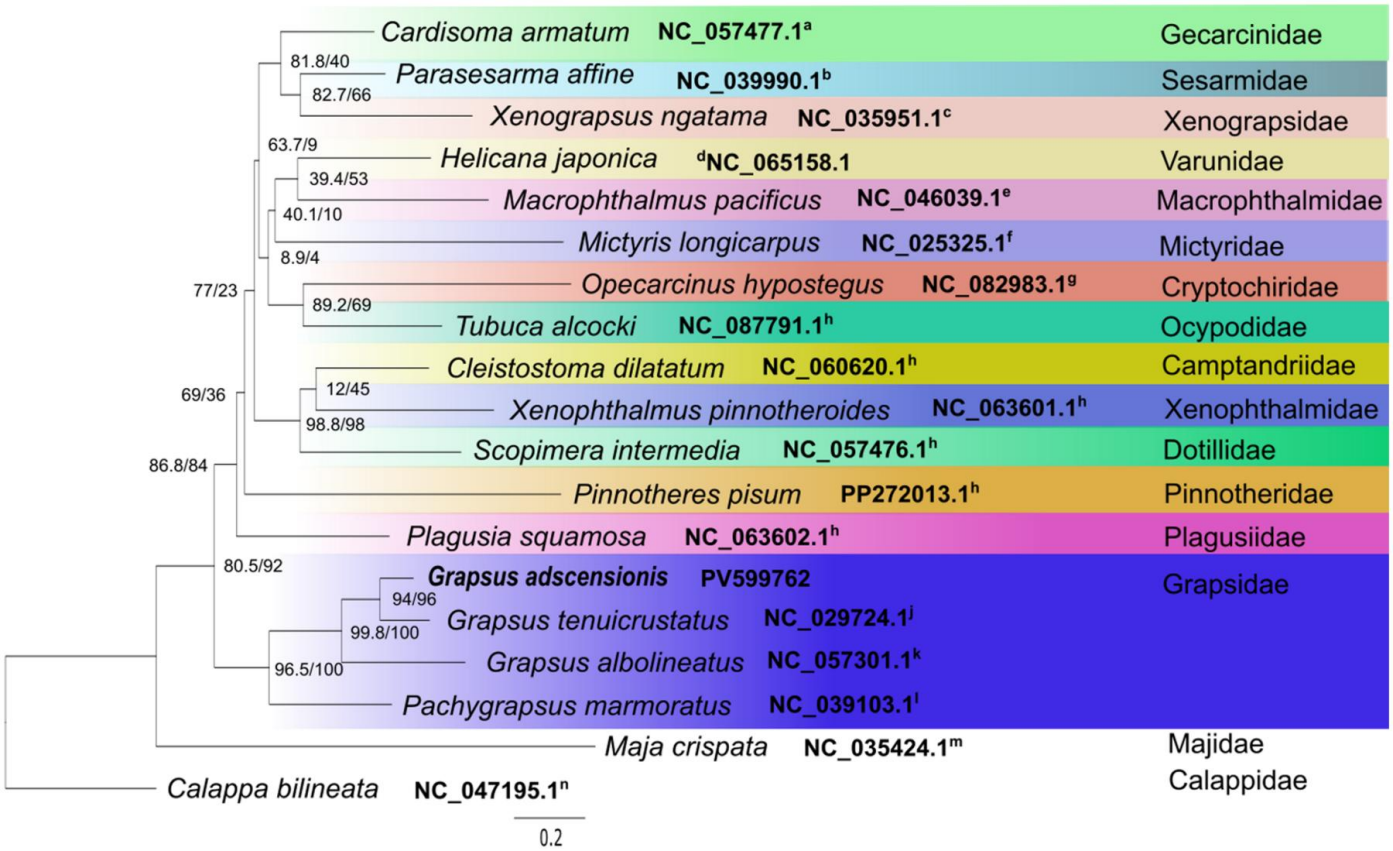
Phylogenetic tree inferred from the nucleotide sequences of 13 PCGs of the mitogenome using ML estimation. Node labels refer to SH-aLRT support (%)/ultrafast bootstrap support (%). Sequences references: ^a^Wang et al. (2021), ^b^Wang, Wang, et al. (2018), ^c^Yang et al. (2008), ^d^Kim et al. (2023), ^e^Wang et al. (2020), ^f^Tan et al. (2016), ^g^Xu et al. (2023), ^h^Unpublished, PV599762, ^j^Sung et al. (2016), ^k^Wang et al. (2022), ^l^Tan et al. (2018), and outgroup (^m^Basso et al. (2017) and ^n^Lu et al. (2020)).

The phylogenetic tree (Figure 3) supports the monophyly of Grapsidae with high bootstrap values, while placing the Plagusiidae family as the closest family. Within the *Grapsus* clade, *G. adscensionis* and *G. tenuicrustatus* (Herbst, 1783) are more closely related to each other than to *G. albolineatus* Latreille in Milbert, 1812.

## Discussion and conclusions

In this work, the complete mitogenome of *G. adscensionis* was sequenced for the first time, and the phylogenetic relationships among Brachyura specimens were analyzed, providing insights into their evolutionary history and genetic divergence. The consistency of the annotations with other decapod mitogenomes, together with the phylogenetic placement in agreement with previous studies, supports the high quality of this mitogenome.

The phylogenetic tree (Figure 3), supports the monophyly of Grapsidae with high bootstrap values, with the Plagusiidae family appearing as the closest family. The close relationship between *G. adscensionis* and *G. tenuicrustatus* in comparison to *G. albolineatus*, may be implying a possible divergence pattern within the genus that could reflect geographic and/or ecological differentiation. Furthermore, the phylogenetic relationships among Macrophthalmidae, Mictyridae, Sesarmidae, Varunidae, and Xenograpsidae are represented and generally align with findings from previous studies (Wang, Ji, et al. 2018; Zhang et al. 2025).

This mitochondrial genome data is an important resource for future phylogenetic and barcoding studies. On one side, the whole mitochondria sequence can be used to better understand the evolutionary and systematic relationships of the Grapsidae family, while on the other side it can serve as basis for developing new markers with multiple applications such as DNA barcoding, metabarcoding and phylogenetics.

## Supplementary Material

Supplementary_Data_CA.docx

## Data Availability

The genome sequence data that support the findings of this study are openly available in GenBank of NCBI (https://www.ncbi.nlm.nih.gov/) under the accession no. PV599762. The associated BioProject, SRA, and Bio-Sample numbers are PRJNA1267061, SRP587244, and SAMN48699784, respectively.
