# Supplementary material for: The mitogenome of the crab Grapsus adscensionis (Osbeck, 1765)
Source: Mitochondrial DNA B Resour. 2025 Oct 16;10(11):1046–9. doi: 10.1080/23802359.2025.2571726 (PMC12532354; doi:10.1080/23802359.2025.2571726)
Supplement: Supplementary_Data_CA.docx [file TMDN_A_2571726_SM4572.docx]

Supplementary material for:

The mitogenome of the crab Grapsus adscensionis (Osbeck, 1765)

**Cátia Alves1,2,3,4,5,6*, Manuel Curto2,6, Lara Baptista1,2,3,5,8, Thapasya Vijayan^7^**, **Lívia Sinigaglia1,2,3,4,5,6, Patrícia Madeira1,2,3,5, António Santos2,4,6, Harald Meimberg7, Manuel Lima2,6 & Sérgio P. Ávila1,2,3,4,5**

1 CIBIO, Centro de Investigação em Biodiversidade e Recursos Genéticos, InBIO Laboratório Associado, Pólo dos Açores, 9501‑801 Ponta Delgada, Portugal

2 BIOPOLIS Program in Genomics, Biodiversity and Land Planning, CIBIO, Campus de Vairão, 4485-661 Vairão, Portugal

3 UNESCO Chair – Land Within Sea: Biodiversity & Sustainability in Atlantic Islands, Universidade dos Açores, R. Mãe de Deus 13A, 9500-321 Ponta Delgada, Portugal

4 Faculdade de Ciências da Universidade do Porto, Rua do Campo Alegre, 1021/1055, 4169‑007 Porto, Portugal

5 MPB - Marine Palaeontology and Biogeography Lab, Universidade dos Açores, Rua da Mãe de Deus, 9501-801 Ponta Delgada, Portugal

6 CIBIO, Centro de Investigação em Biodiversidade e Recursos Genéticos, InBIO Laboratório Associado, Universidade do Porto, Campus de Vairão, Rua Padre Armando Quintas nº 7, 4485-661 Vairão

7 BOKU University, Department of Ecosystem Management, Climate and Biodiversity, Institute of Integrative Nature Conservation Research, Vienna, Austria

_8_ Royal Netherlands Institute for Sea Research, Landsdiep 4, 1797 SZ ‘t Hoorntje, Texel, Netherlands

*catia.alves@cibio.up.pt

1. **DNA extraction protocol**

**List of reagents:**

- Lysis buffer (2% SDS, 2% PVP 40, 250 Mm NaCl, 200 Mm Tris HCl, 5 Mm EDTA, pH= 8.0)
- Proteinase K (10 mg/mL)
- RNase (10 mg/mL)
- 3 M potassium acetate (pH=4.7)
- Binding buffer (Composition: 237.5 ml EtOH absolute, 12.5 mL H₂0, 47.756 g guanidine hydrochloride, pH= 5.5)
- Elution buffer (10 Nm Tris, pH= 8.0)

**Protocol:**

The crab leg samples were dissected, and approximately 0.5 cm of muscle tissue was removed from each specimen. The tissue samples were then placed in Eppendorf tubes and briefly exposed to air to allow residual ethanol to evaporate.

**1)** 500 µL of lysis buffer + 16 µL of Proteinase K added to each sample. The solution was shortly vortexed and left in a thermomixer overnight (56 °C, 300 rpm).

**2)** 16 µL of RNase added to the solution, vortexed and incubated for 15 minutes in the thermomixer (37 °C, 300 rpm). 125 µL of potassium acetate (KOAc), - stored at -20 °C-, added to the solution.

**3)** Stepwise centrifugation (ThermoScientific, Heraeus Multifuge X3R Centrifuge) as follows: 1000 rpm (1 min), 2000 rpm (1 min), 4000 rpm (1 min), 8000 rpm (1 min), 11000 rpm (7 min).

**4)** 500 µL of supernatant was transferred to a 96 well, 2 mL deepwell plate containing 750 µL of binding buffer. The solution was mixed 10 times and 500 µL were transferred to a 96 well silica membrane EconoSpin® plate.

**5)** Stepwise centrifugation as follows: 500 rpm (2 min), 4000 rpm (5 min), 6000 rpm (5 min); flow-through solution was discarded.

**6)** Steps 4 and 5 repeated with the remaining solution.

**7)** Two ethanol washing-steps were conducted: in each step 600 µL of 80% ethanol were added to the EconoSpin® plate and centrifuged first at 4000 rpm (2 min), flow-through was discarded; next centrifugation at 6000 rpm (2 min).

**8)** Ethanol traces were removed by drying the EconoSpin® plate for 10 min at room temperature to ensure its complete evaporation.

**9)** Elution on a 96-well plate: first elution step by adding 50 µL of elution buffer and centrifuging at 6000 rpm (3 min). Second elution step in a new 96-well plate by adding 50 µL of Elution buffer and centrifuging at 6000 rpm (3 min). Both flow-through solutions containing DNA were preserved at -20 °C.

1. **Genome assembly report**


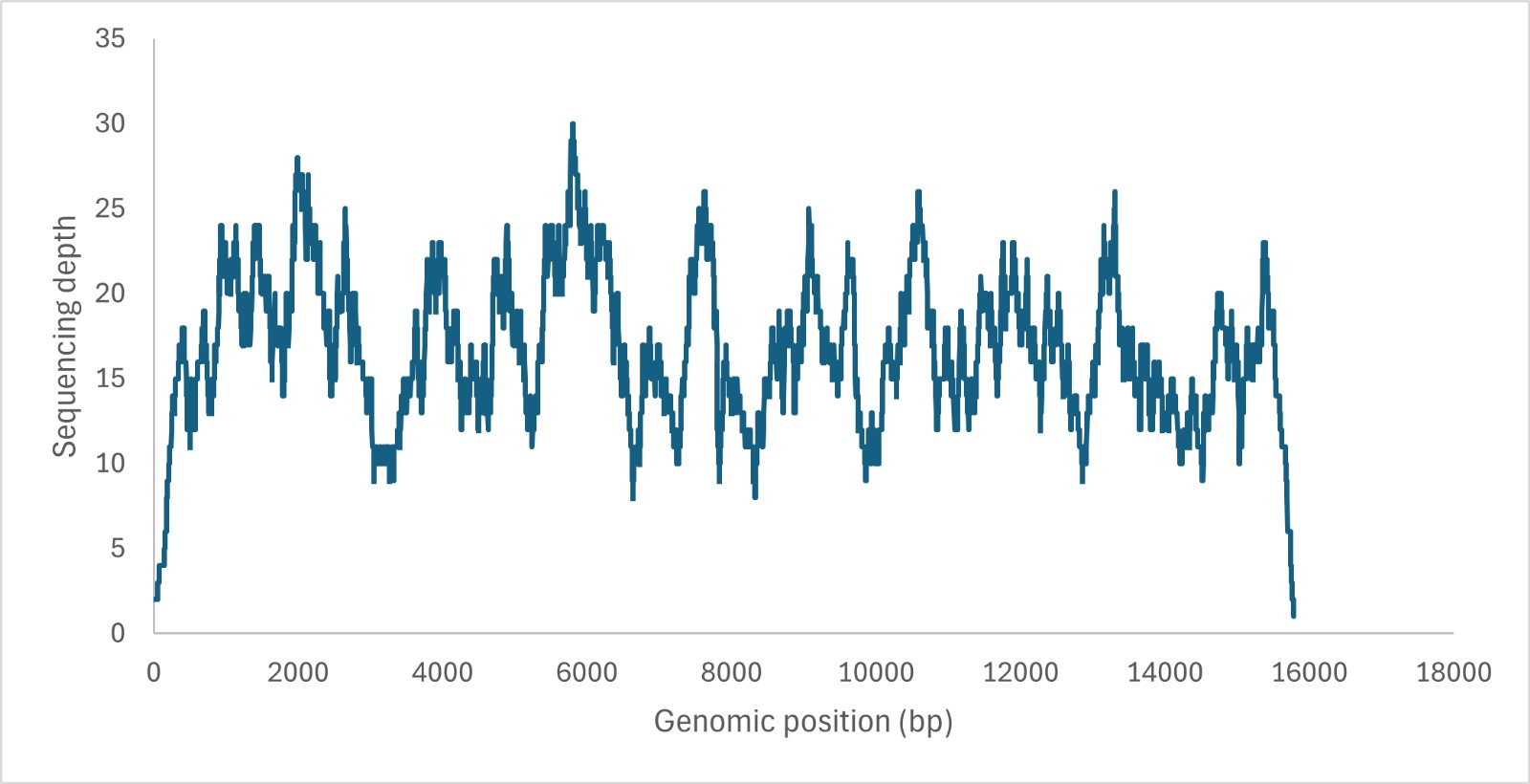


**Figure S1** - The read coverage depth map of the assembly of the mitogenome of Grapsus adscensionis.

1. **Annotation of the complete mitochondrial genome of *Grapsus adscensionis***

**Table S1** - Annotation of the complete mitochondrial genome of Grapsus adscensionis

| **Gene** | **Position**  From | To | **Length** | **Amino acid** | **Start/stop codon** | **Anticodon** | **Intergenic region** | **Strand** |
| --- | --- | --- | --- | --- | --- | --- | --- | --- |
| *cox1* | 1 | 1539 | 1539 | 513 | ATG/TAA |  | 0 | H |
| trnl2 | 1,535 | 1,603 | 68 |  |  | TAA | -3 | H |
| *cox2* | 1607 | 2320 | 713 | 238 | ATG/GCT |  | 5 | H |
| trnK | 2295 | 2363 | 68 |  |  | TTT | 2 | H |
| trnD | 2364 | 2426 | 62 |  |  | GTC | 3 | H |
| *atp8* | 2428 | 2586 | 158 | 53 | ATA/TAA |  | -5 | H |
| *atp6* | 2580 | 3254 | 674 | 225 | ATA/TAA |  | 1 | H |
| *cox3* | 3254 | 4045 | 791 | 264 | ATG/TGA |  | 1 | H |
| trnG | 4045 | 4107 | 62 |  |  | TCC | -1 | H |
| *ND3* | 4105 | 4458 | 353 | 118 | ATA/TAA |  | 3 | H |
| trnA | 4460 | 4526 | 66 |  |  | TGC | 5 | H |
| trnR | 4530 | 4593 | 63 |  |  | TCG | 2 | H |
| trnN | 4594 | 4660 | 66 |  |  | GTT | 7 | H |
| trnS1 | 4666 | 4732 | 66 |  |  | TCT | 4 | H |
| trnE | 4735 | 4802 | 67 |  |  | TTC | 69 | H |
| trnH | 4806 | 4870 | 64 |  |  | GTG | 132 | L |
| trnF | 4873 | 4937 | 64 |  |  | GAA | 51 | L |
| *nad5* | 4987 | 6717 | 1730 | 577 | ATT/TAA |  | 45 | L |
| *nad4* | 6761 | 8098 | 1337 | 446 | ATG/TAG |  | -5 | L |
| *nad4L* | 8092 | 8394 | 302 | 101 | ATG/TAA |  | 29 | L |
| trnT | 8422 | 8487 | 65 |  |  | TGT | 68 | H |
| trnP | 8488 | 8554 | 66 |  |  | TGG | 76 | L |
| *nad6* | 8563 | 9060 | 497 | 166 | ATA/TAA |  | 1 | H |
| *cob* | 9060 | 10194 | 1134 | 394 | ATA/TAA |  | -45 | H |
| trnS2 | 10197 | 10261 | 64 |  |  | TGA | 24 | H |
| *nad1* | 10284 | 11231 | 947 | 316 | ATT/TAA |  | 93 | L |
| trnL1 | 11256 | 11323 | 67 |  |  | TAG | 64 | L |
| rrnL | 11319 | 12659 | 1340 | 447 |  |  | 63 | L |
| trnV | 12649 | 12721 | 72 |  |  | TAC | 74 | L |
| rrnS | 12722 | 13550 | 828 | 276 |  |  | 2 | L |
| OL | 13551 | 14129 | 578 |  |  |  | 3 | L |
| trnI | 14131 | 14196 | 65 |  |  | GAT | 67 | H |
| trnQ | 14194 | 14262 | 68 |  |  | TTG | 79 | L |
| trnM | 14272 | 14342 | 70 |  |  | CAT | 2 | H |
| *nad2* | 14343 | 15353 | 1010 | 337 | ATG/TAG |  | -3 | H |
| rnW | 15352 | 15420 | 68 |  |  | TCA | 6 | H |
| trnC | 15425 | 15488 | 63 |  |  | GCA | 66 | L |
| trnY | 15489 | 15553 | 64 | 513 |  | GTA | 0 | L |
